# Supplementary material for: A curated human cellular microRNAome based on 196 primary cell types
Source: Gigascience. 2022 Aug 25;11:giac083. doi: 10.1093/gigascience/giac083 (PMC9404528; doi:10.1093/gigascience/giac083)
Supplement: giac083_Supplemental_Files [file giac083_supplemental_files.zip › Supplementary_Figure_S11_Stem.pdf]

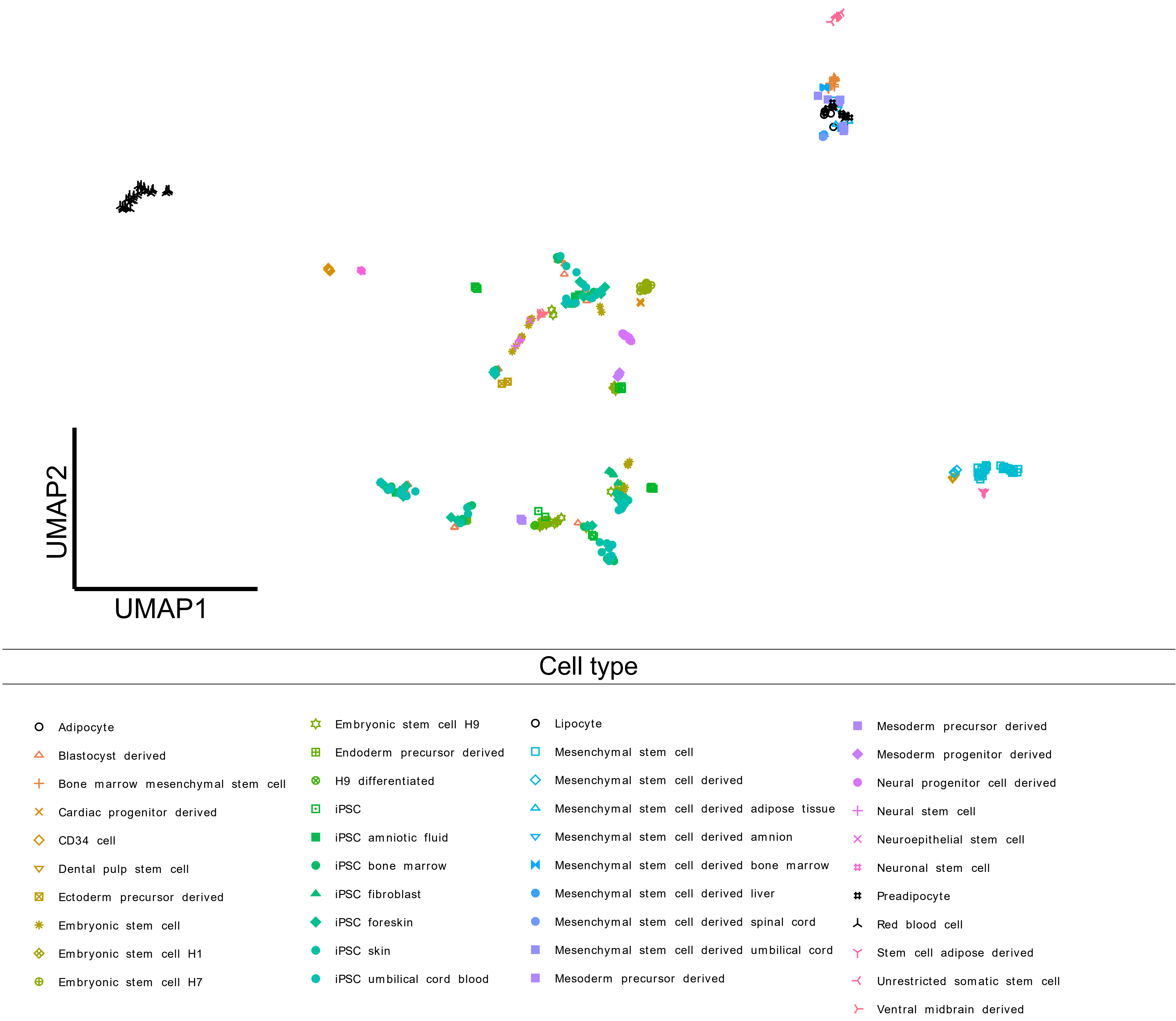
UMAP2
UMAP1
Cell type
Adipocyte
Blastocyst derived
Bone marrow mesenchymal stem cell
Cardiac progenitor derived
CD34 cell
Dental pulp stem cell
Ectoderm precursor derived
Embryonic stem cell
Embryonic stem cell H1
Embryonic stem cell H7
Embryonic stem cell H9
Endoderm precursor derived
H9 differentiated
iPSC
iPSC amniotic fluid
iPSC bone marrow
iPSC fibroblast
iPSC foreskin
iPSC skin
iPSC umbilical cord blood
Lipocyte
Mesenchymal stem cell
Mesenchymal stem cell derived
Mesenchymal stem cell derived adipose tissue
Mesenchymal stem cell derived amnion
Mesenchymal stem cell derived bone marrow
Mesenchymal stem cell derived liver
Mesenchymal stem cell derived spinal cord
Mesenchymal stem cell derived umbilical cord
Mesoderm precursor derived
Mesoderm precursor derived
Mesoderm progenitor derived
Neural progenitor cell derived
Neural stem cell
Neuroepithelial stem cell
Neuronal stem cell
Preadipocyte
Red blood cell
Stem cell adipose derived
Unrestricted somatic stem cell
Ventral midbrain derived
