# Supplementary material for: A curated human cellular microRNAome based on 196 primary cell types
Source: Gigascience. 2022 Aug 25;11:giac083. doi: 10.1093/gigascience/giac083 (PMC9404528; doi:10.1093/gigascience/giac083)
Supplement: giac083_Supplemental_Files [file giac083_supplemental_files.zip › Supplementary_Figure_S2_Endothelial.pdf]

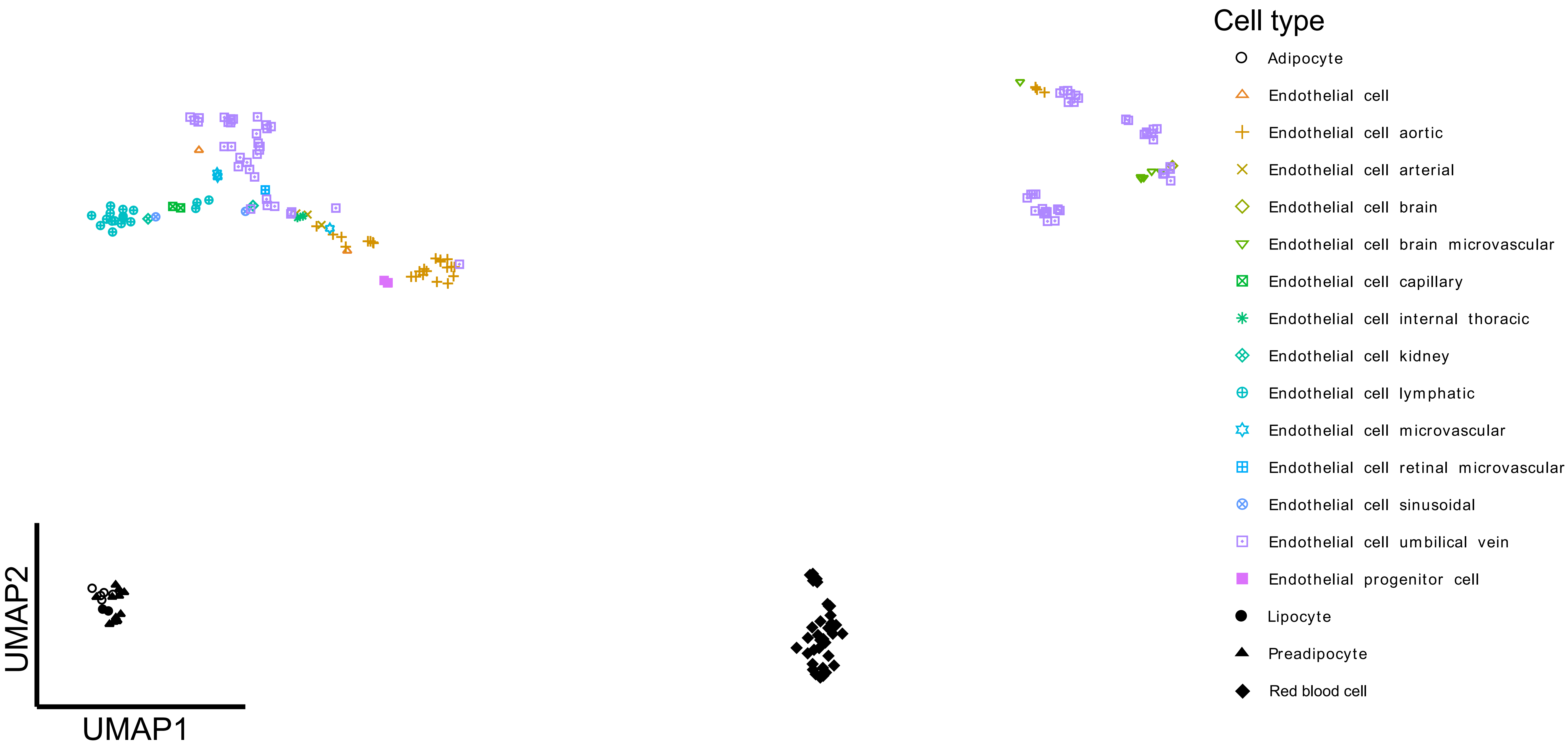
UMAP2
UMAP1
Cell type
Adipocyte
Endothelial cell
Endothelial cell aortic
Endothelial cell arterial
Endothelial cell brain
Endothelial cell brain microvascular
Endothelial cell capillary
Endothelial cell internal thoracic
Endothelial cell kidney
Endothelial cell lymphatic
Endothelial cell microvascular
Endothelial cell retinal microvascular
Endothelial cell sinusoidal
Endothelial cell umbilical vein
Endothelial progenitor cell
Lipocyte
Preadipocyte
Red blood cell
