# Supplementary material for: A curated human cellular microRNAome based on 196 primary cell types
Source: Gigascience. 2022 Aug 25;11:giac083. doi: 10.1093/gigascience/giac083 (PMC9404528; doi:10.1093/gigascience/giac083)
Supplement: giac083_Supplemental_Files [file giac083_supplemental_files.zip › Supplementary_Figure_S3_Epithelial.pdf]

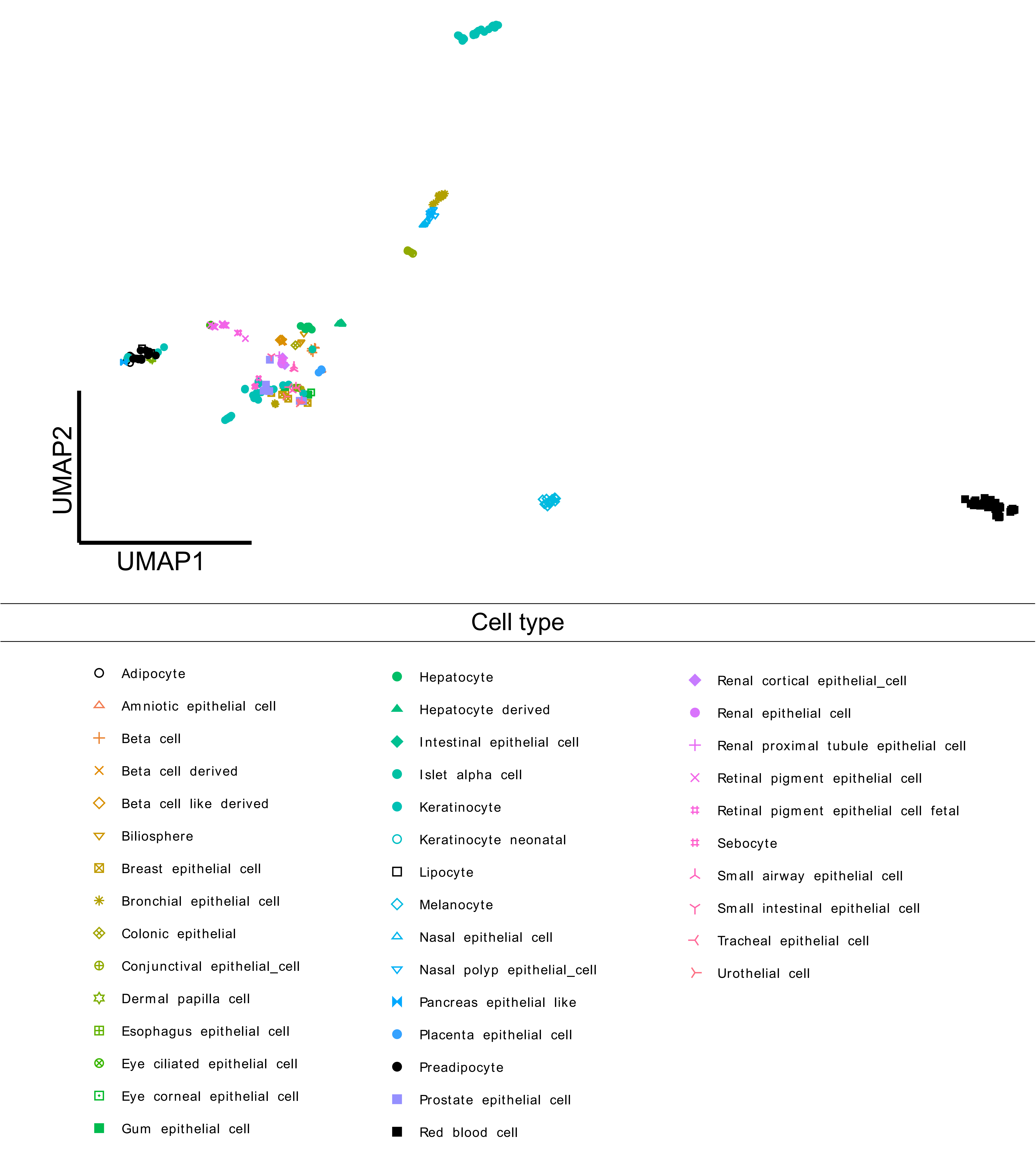

UMAP2
UMAP1
Cell type
Adipocyte
Amniotic epithelial cell
Beta cell
Beta cell derived
Beta cell like derived
Biliosphere
Breast epithelial cell
Bronchial epithelial cell
Colonic epithelial
Conjunctival epithelial_cell
Dermal papilla cell
Esophagus epithelial cell
Eye ciliated epithelial cell
Eye corneal epithelial cell
Gum epithelial cell
Hepatocyte
Hepatocyte derived
Intestinal epithelial cell
Islet alpha cell
Keratinocyte
Keratinocyte neonatal
Lipocyte
Melanocyte
Nasal epithelial cell
Nasal polyp epithelial_cell
Pancreas epithelial like
Placenta epithelial cell
Preadipocyte
Prostate epithelial cell
Red blood cell
Renal cortical epithelial_cell
Renal epithelial cell
Renal proximal tubule epithelial cell
Retinal pigment epithelial cell
Retinal pigment epithelial cell fetal
Sebocyte
Small airway epithelial cell
Small intestinal epithelial cell
Tracheal epithelial cell
Urothelial cell
