# Supplementary material for: A curated human cellular microRNAome based on 196 primary cell types
Source: Gigascience. 2022 Aug 25;11:giac083. doi: 10.1093/gigascience/giac083 (PMC9404528; doi:10.1093/gigascience/giac083)
Supplement: giac083_Supplemental_Files [file giac083_supplemental_files.zip › Supplementary_Figure_S4_Fibroblasts.pdf]

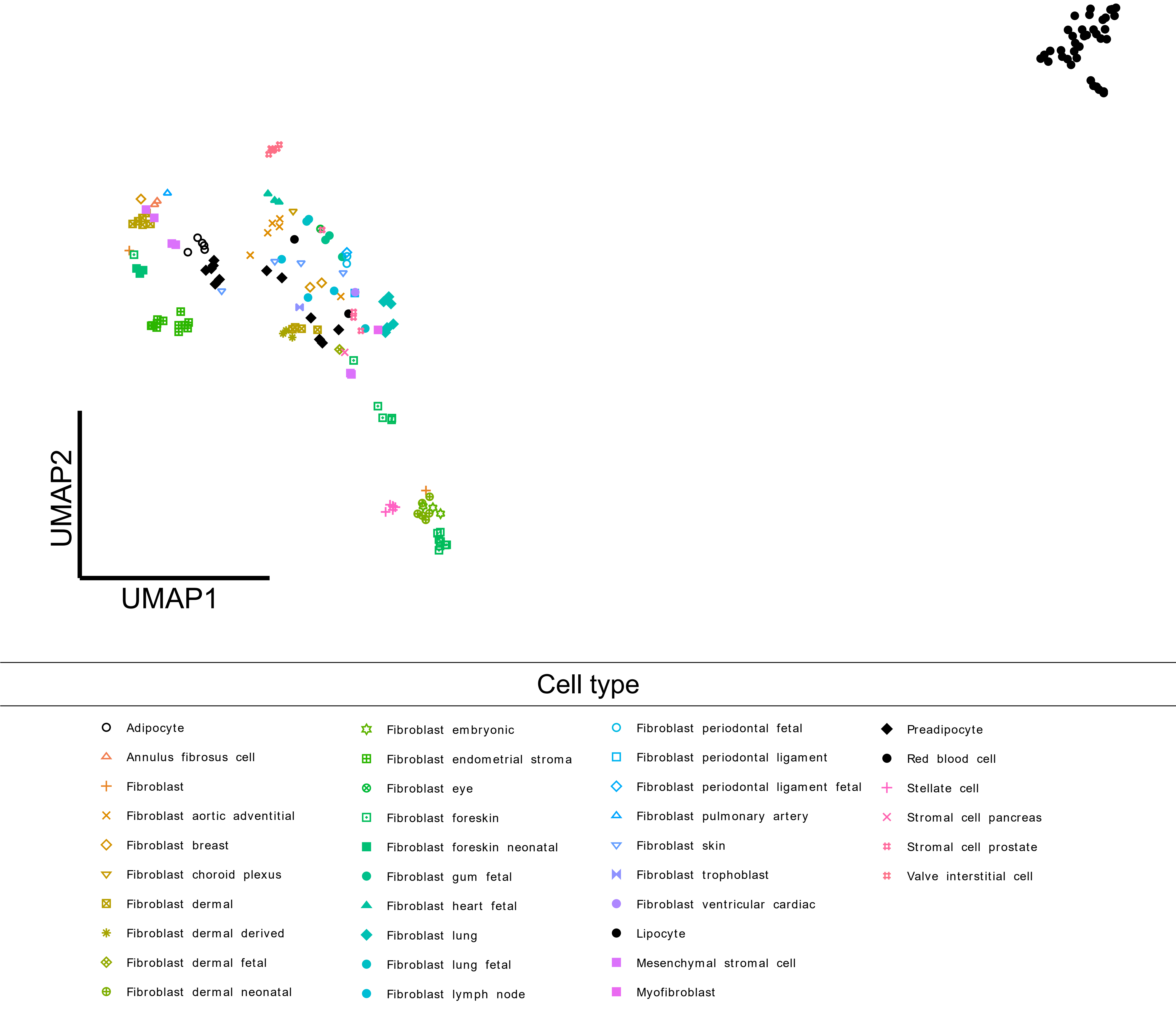
UMAP2
UMAP1
Cell type
Adipocyte
Annulus fibrosus cell
Fibroblast
Fibroblast aortic adventitial
Fibroblast breast
Fibroblast choroid plexus
Fibroblast dermal
Fibroblast dermal derived
Fibroblast dermal fetal
Fibroblast dermal neonatal
Fibroblast embryonic
Fibroblast endometrial stroma
Fibroblast eye
Fibroblast foreskin
Fibroblast foreskin neonatal
Fibroblast gum fetal
Fibroblast heart fetal
Fibroblast lung
Fibroblast lung fetal
Fibroblast lymph node
Fibroblast periodontal fetal
Fibroblast periodontal ligament
Fibroblast periodontal ligament fetal
Fibroblast pulmonary artery
Fibroblast skin
Fibroblast trophoblast
Fibroblast ventricular cardiac
Lipocyte
Mesenchymal stromal cell
Myofibroblast
Preadipocyte
Red blood cell
Stellate cell
Stromal cell pancreas
Stromal cell prostate
Valve interstitial cell
