# Supplementary material for: A curated human cellular microRNAome based on 196 primary cell types
Source: Gigascience. 2022 Aug 25;11:giac083. doi: 10.1093/gigascience/giac083 (PMC9404528; doi:10.1093/gigascience/giac083)
Supplement: giac083_Supplemental_Files [file giac083_supplemental_files.zip › Supplementary_Figure_S5_Immune.pdf]

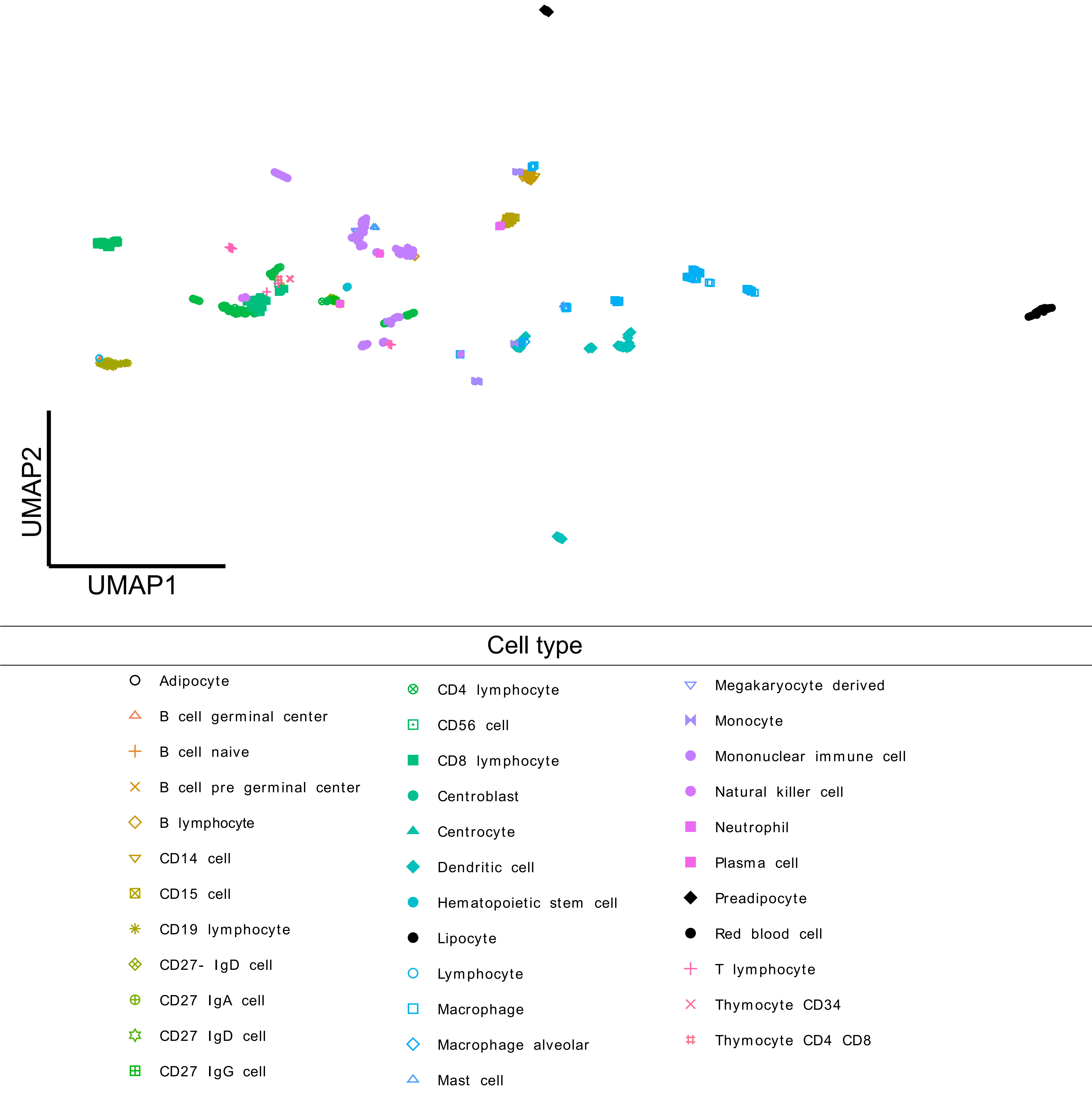
UMAP2
UMAP1
Cell type
Adipocyte
B cell germinal center
B cell naive
B cell pre germinal center
B lymphocyte
CD14 cell
CD15 cell
CD19 lymphocyte
CD27- IgD cell
CD27 IgA cell
CD27 IgD cell
CD27 IgG cell
CD4 lymphocyte
CD56 cell
CD8 lymphocyte
Centroblast
Centrocyte
Dendritic cell
Hematopoietic stem cell
Lipocyte
Lymphocyte
Macrophage
Macrophage alveolar
Mast cell
Megakaryocyte derived
Monocyte
Mononuclear immune cell
Natural killer cell
Neutrophil
Plasma cell
Preadipocyte
Red blood cell
T lymphocyte
Thymocyte CD34
Thymocyte CD4 CD8
