# Supplementary material for: A curated human cellular microRNAome based on 196 primary cell types
Source: Gigascience. 2022 Aug 25;11:giac083. doi: 10.1093/gigascience/giac083 (PMC9404528; doi:10.1093/gigascience/giac083)
Supplement: giac083_Supplemental_Files [file giac083_supplemental_files.zip › Supplementary_Figure_S6_Muscle.pdf]

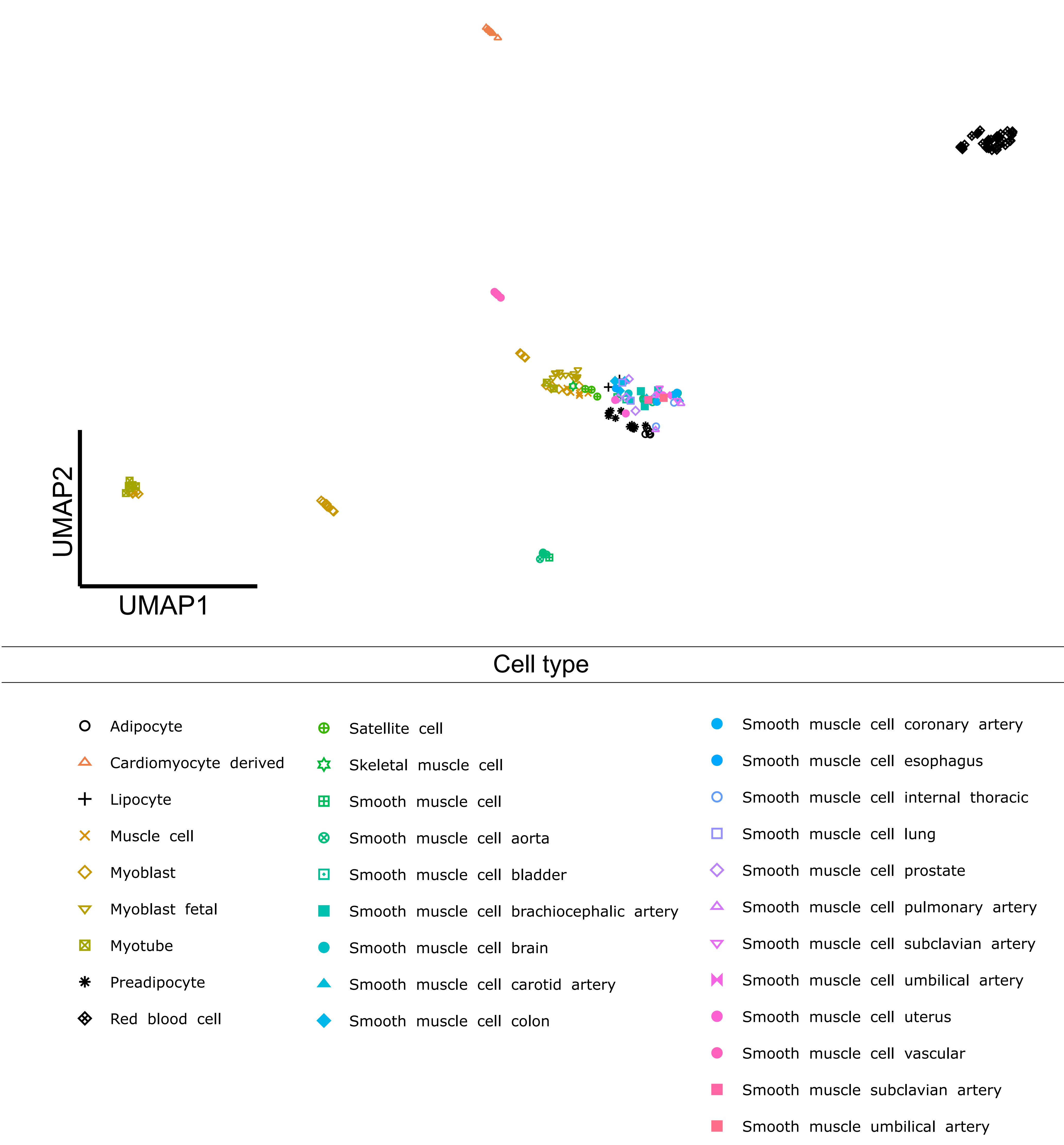
UMAP2
UMAP1
Cell type
Adipocyte
Cardiomyocyte derived
Lipocyte
Muscle cell
Myoblast
Myoblast fetal
Myotube
Preadipocyte
Red blood cell
Satellite cell
Skeletal muscle cell
Smooth muscle cell
Smooth muscle cell aorta
Smooth muscle cell bladder
Smooth muscle cell brachiocephalic artery
Smooth muscle cell brain
Smooth muscle cell carotid artery
Smooth muscle cell colon
Smooth muscle cell coronary artery
Smooth muscle cell esophagus
Smooth muscle cell internal thoracic
Smooth muscle cell lung
Smooth muscle cell prostate
Smooth muscle cell pulmonary artery
Smooth muscle cell subclavian artery
Smooth muscle cell umbilical artery
Smooth muscle cell uterus
Smooth muscle cell vascular
Smooth muscle subclavian artery
Smooth muscle umbilical artery
