# Supplementary material for: A curated human cellular microRNAome based on 196 primary cell types
Source: Gigascience. 2022 Aug 25;11:giac083. doi: 10.1093/gigascience/giac083 (PMC9404528; doi:10.1093/gigascience/giac083)
Supplement: giac083_Supplemental_Files [file giac083_supplemental_files.zip › Supplementary_Figure_S7_Other.pdf]

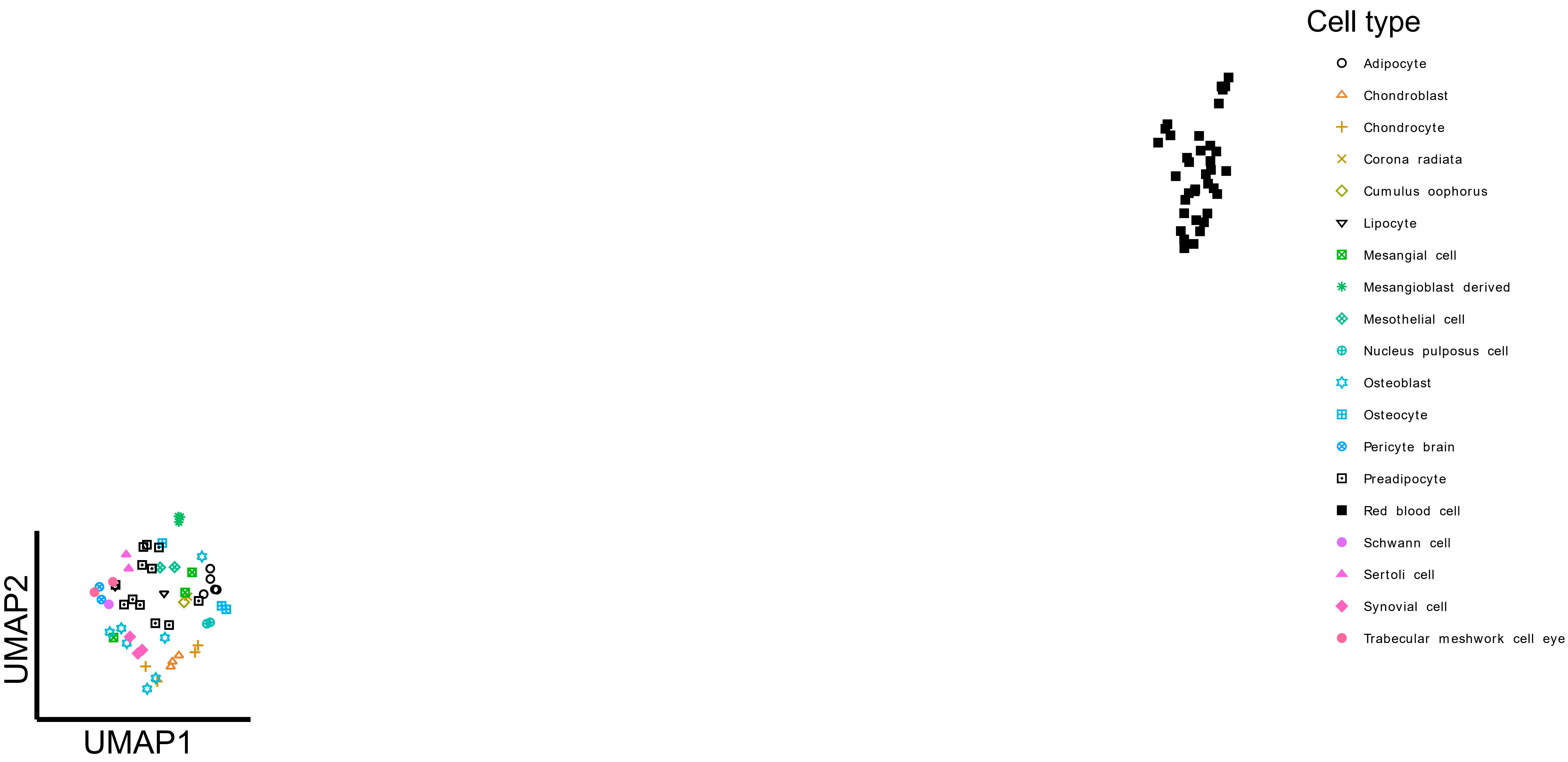
UMAP2
UMAP1
Cell type
Adipocyte
Chondroblast
Chondrocyte
Corona radiata
Cumulus oophorus
Lipocyte
Mesangial cell
Mesangioblast derived
Mesothelial cell
Nucleus pulposus cell
Osteoblast
Osteocyte
Pericyte brain
Preadipocyte
Red blood cell
Schwann cell
Sertoli cell
Synovial cell
Trabecular meshwork cell eye
