# Supplementary figures and images for: A curated human cellular microRNAome based on 196 primary cell types
Source: Gigascience. 2022 Aug 25;11:giac083. doi: 10.1093/gigascience/giac083 (PMC9404528; doi:10.1093/gigascience/giac083)

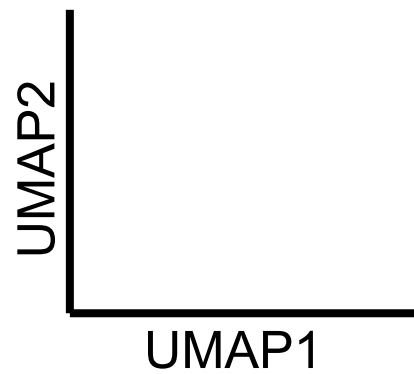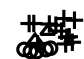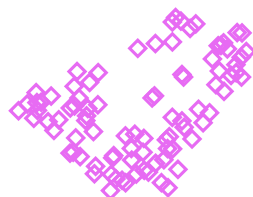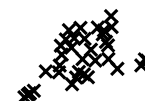

## Cell type

- Adipocyte
- △ Lipocyte
- + Preadipocyte
- × Red blood cell
- ◇ Sperm

Supplement: giac083_Supplemental_Files [file giac083_supplemental_files.zip › Supplementary_Figure_S10_Sperm.pdf]

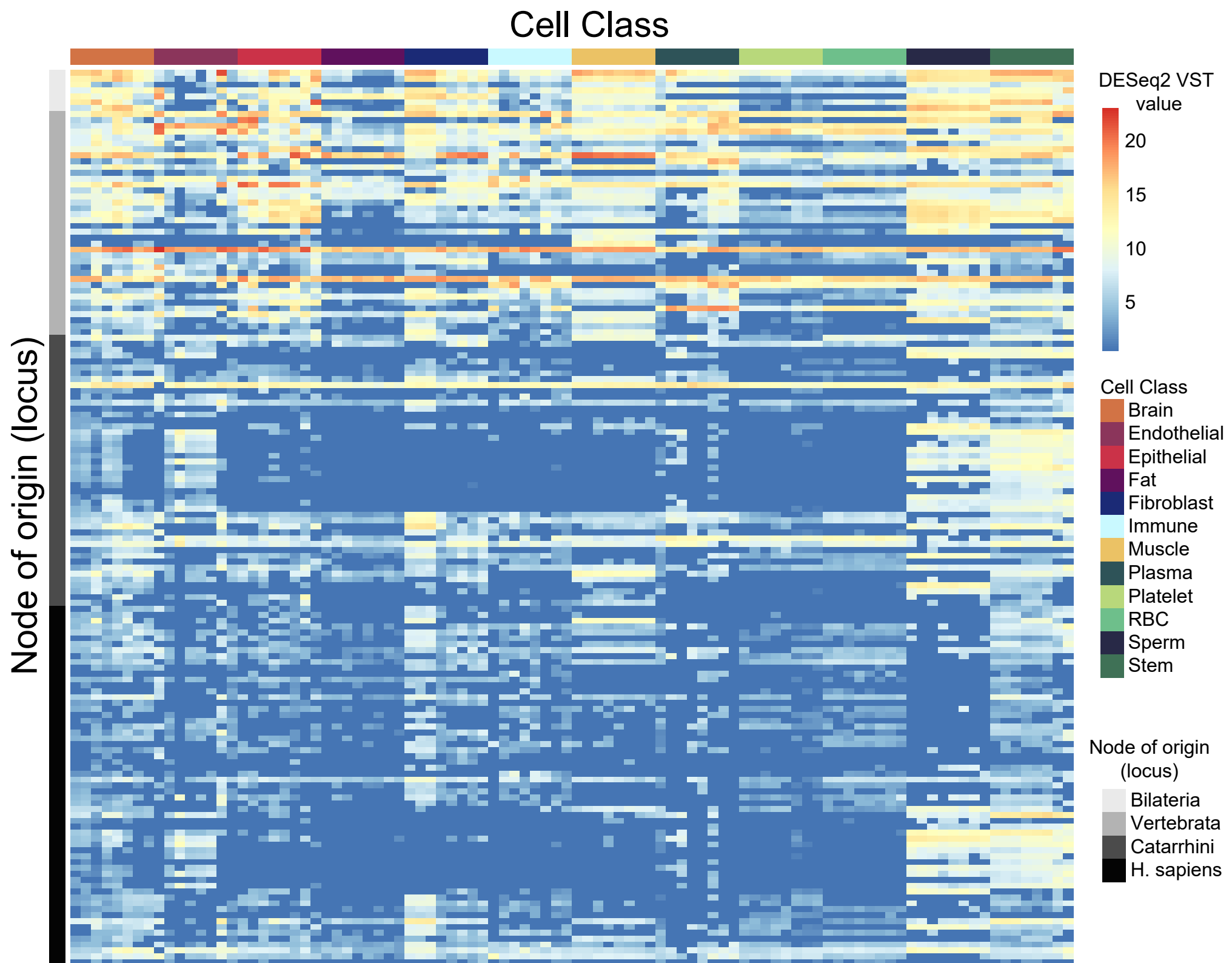

Supplement: giac083_Supplemental_Files [file giac083_supplemental_files.zip › Supplementary_Figure_S12_Evolution.pdf]

chr17 (p13.3) 17p13.3 p13.2 17p13.1 17p12 17p11.2 17q11.2 17q12 q21.2 17q21.31 21.32 q21.33 17q22 q23.2 q24.2 17q24.3 17q25.1 17q25.3

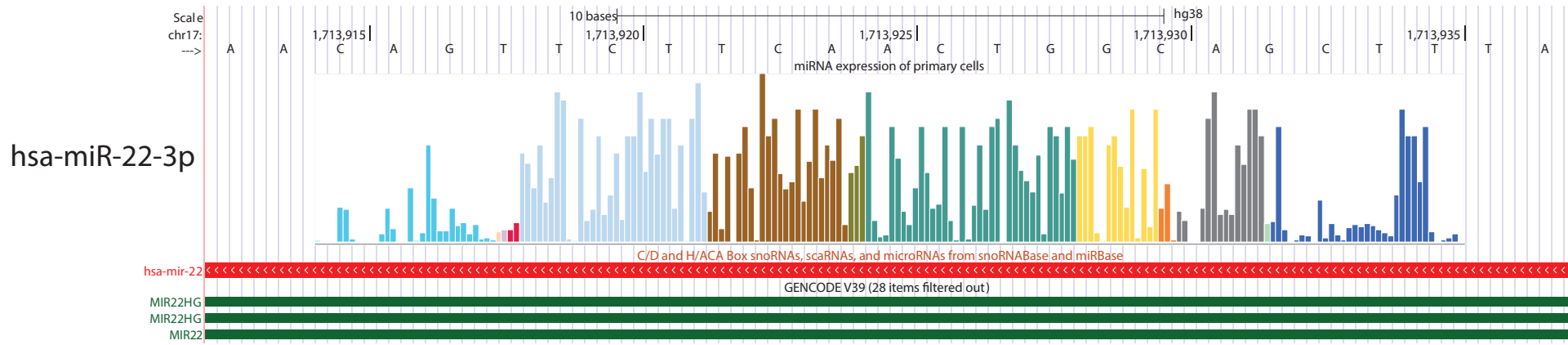

B

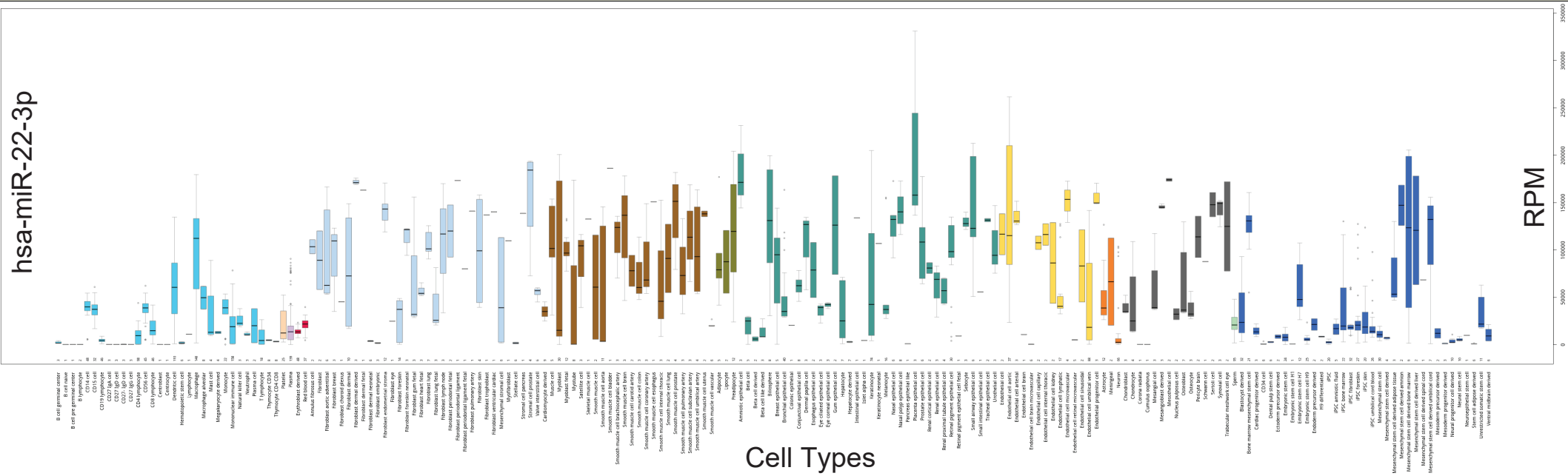

Supplement: giac083_Supplemental_Files [file giac083_supplemental_files.zip › Supplementary_Figure_S13_GenomeBrowser.pdf]

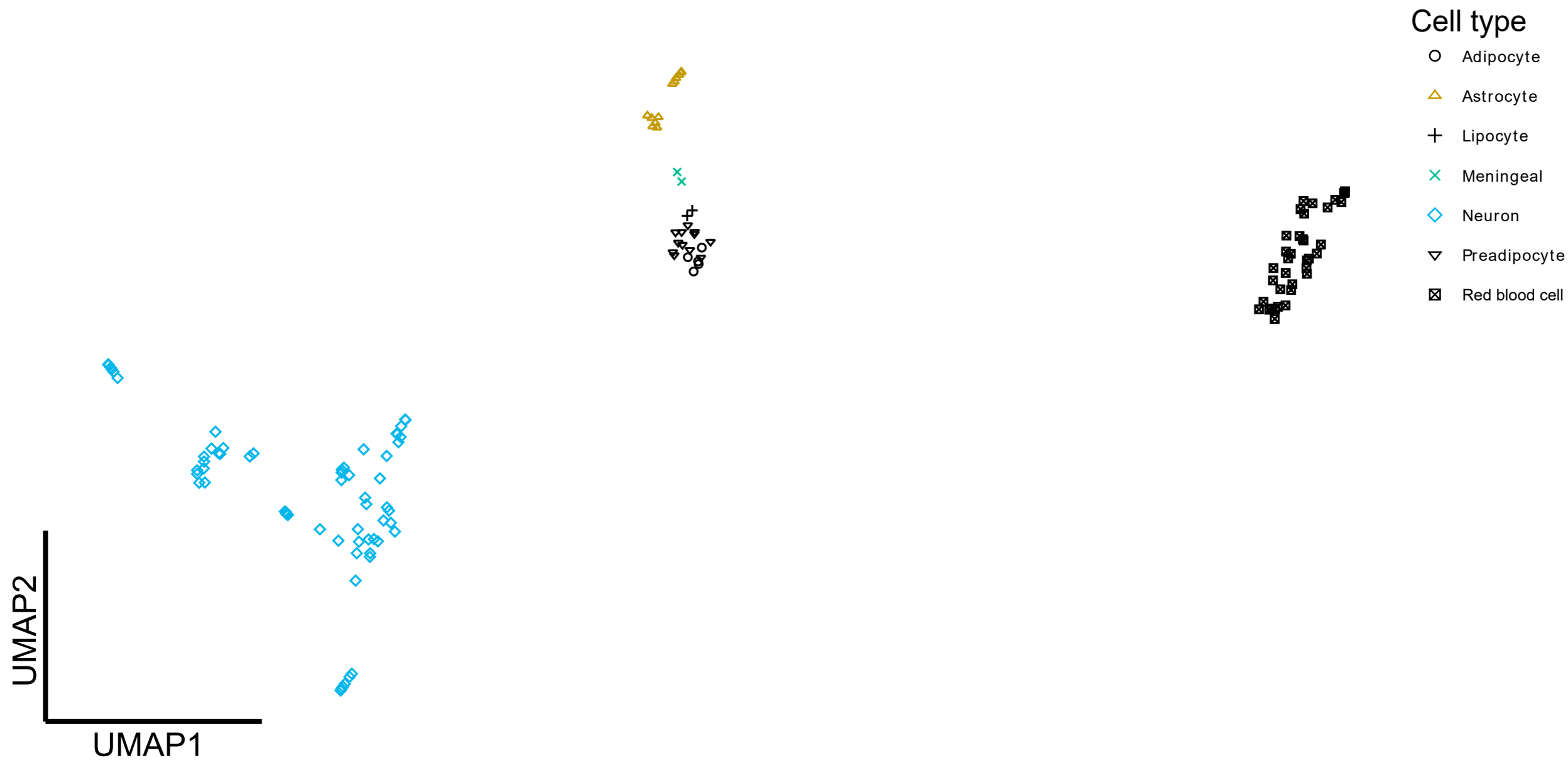

Supplement: giac083_Supplemental_Files [file giac083_supplemental_files.zip › Supplementary_Figure_S1_Brain.pdf]

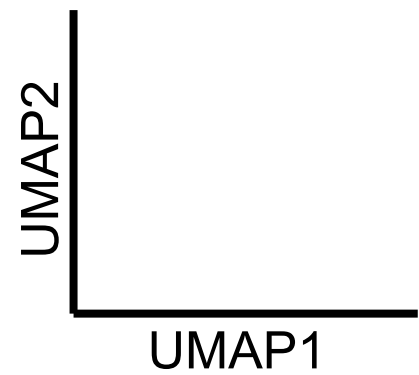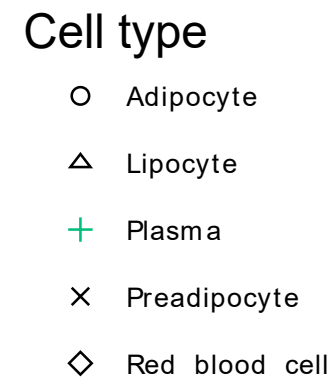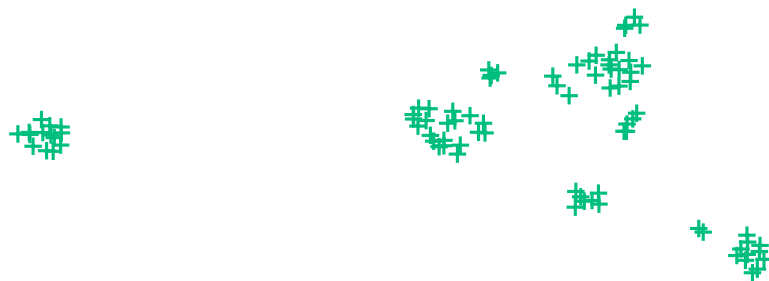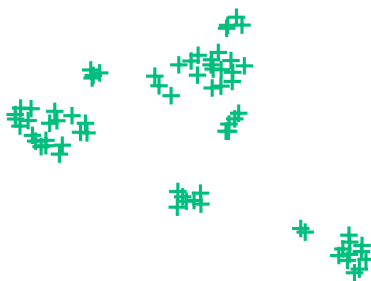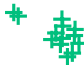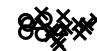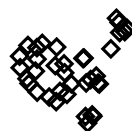

Supplement: giac083_Supplemental_Files [file giac083_supplemental_files.zip › Supplementary_Figure_S8_Plasma.pdf]

# Cell type

- Adipocyte
- △ Lipocyte
- + Platelet
- × Preadipocyte
- ◇ Red blood cell

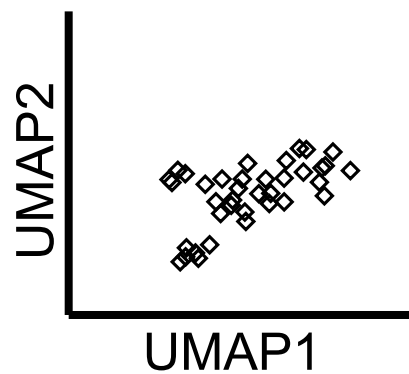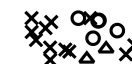

Supplement: giac083_Supplemental_Files [file giac083_supplemental_files.zip › Supplementary_Figure_S9_Platelet.pdf]
