# Supplementary material for: A curated human cellular microRNAome based on 196 primary cell types
Source: Gigascience. 2022 Aug 25;11:giac083. doi: 10.1093/gigascience/giac083 (PMC9404528; doi:10.1093/gigascience/giac083)
Supplement: giac083_Response_to_Reviewer_Comments_Original_Submission [file giac083_response_to_reviewer_comments_original_submission.pdf]

Author's Response To Reviewer Comments

Close

See the file as uploaded as a supplemental material.

Close
