# Supplementary material for: A curated human cellular microRNAome based on 196 primary cell types
Source: Gigascience. 2022 Aug 25;11:giac083. doi: 10.1093/gigascience/giac083 (PMC9404528; doi:10.1093/gigascience/giac083)
Supplement: giac083_Reviewer_3_Report_Original_Submission — Kevin Peterson -- 6/2/2022 Reviewed [file giac083_reviewer_3_report_original_submission.pdf]

Reviewer Report

Title: A curated human cellular microRNAome based on 196 primary cell types

Version: Original Submission **Date:** 6/2/2022

Reviewer name: Kevin Peterson

Reviewer Comments to Author:

Pati et al. report the expression profiles of miRNAs and a vast array of other, non-miRNA sequences, across 196 cell types based on thousands of publicaly available data sets. Although this will be an outstanding contribution to the miRNA field, I am recommending rejection for now with the strong encouragement to resubmit once the authors have addressed my concerns. To be frank, the authors have two diametrically opposed research agendas here: 1) What are the expression profiles of bona fide miRNAs (as determined by MirGeneDB); and 2) What else might be expressed in human cell types that could be of interest to small RNA workers and clinicians? Because of these opposed goals, the paper is not only confusing to read and process, but it gives fodder to the numerous paper-mill products that continue to identify non-miRNAs as diagnostic, prognostic, and even mechanistic indicators into virtually every human malady under the sun.

Let me try and highlight why the use of only MirGeneDB (MGDB) would be highly useful for this paper.

1) miRBase (MB) is not consistent in its identification of both arms of a bona fide hairpin, resulting in the authors not reporting star reads for highly expressed miRNAs such as mir-206 and mir-184. Further, there are numerous examples where the authors do in fact report a "mature" versus "star" read without both arms annotated in MB with some included in MGDB (e.g., Mir-944, Mir-3909) and others not (e.g., mir-3615) raising the question of how these data were annotated.

2) The authors write that, "the majority (46%) of the reads are mature miRNAs." But MB makes no attempt to distinguish mature from star arms. Hence, if they are annotating to MB, they cannot distinguish between these two processing products. This is not only confusing, but also very unfortunate as one cannot get a sense of the expression of evolutionary intended gene products versus processing products.

3) The authors report on the use of 5p versus 3p strand dominance, but have no examples of "co-dominant" miRNAs (Fig. 1C) when, in fact, there are numerous examples in their data including Mir-324, Mir-300, Mir-339, Mir-361 etc. with some switching arms depending on the variant. All of this is available at MGDB; none at MB.

3) MB does not allow the identification of loop or offset reads separate from the arm reads, allowing to authors to accurately report the amount of reads derived from the "hairpin" versus the arms (and how the authors reported this in Fig. 1B is not at all clear given that these sequences are not annotated as such at MB).

4) The authors bias their genic origins of small RNA reads by filtering first using MB, and then identifying remaining reads as arising from other sources including tRNAs, rRNAs, mRNAs etc. However, numerous "miRNAs" in MB arise from these genic sources including mir-484 (mRNA) and mir-3648 (rRNA). So if I understand the authors pipe-line these sequences are mistakenly included in the "mature miRNA"

column.
5) The use of MGDB would allow the user to see the saturation of mature reads across the different cell types in Fig. 1E, and, if mature is distinguished by star, then one could also see the (near)-saturation of star reads as well. As it stands, their plot just simply highlights the non-genic nature of much of MB. Further, because MGDB identifies the age of each miRNA, if the authors were interested, they could also test a long-standing pattern that evolutionary older miRNAs are expressed at higher levels than younger miRNAs relative to specific cell types.
6) The authors report the expression profiles of bona fide miRNAs in Figs 3 and 5, but report the expression profiles of non-miRNAs in Fig. 4. These include mir-3150b, mir-4298, mir-569, mir-934, mir-302f, and mir-663b. None of these supposed miRNAs have the requisite reads for miRNA annotation, and all but mir-3150b fail a structural examination as well. In fact, MGDG has no reads (which includes numerous data sets from the Halushka lab) for mir-302f, mi-4298, and mir-569, and only a few reads from one "arm" for mir-663b and mir-3150, highlighting the need to examine these supposed reads in detail. The inclusion of obvious non-miRNAs here is confusing and needlessly undermines the authors study and conclusions.
So, my strong recommendation is to potentially write two papers. The first (this one here) focuses only on the expression of miRNAs, emphasizing really interesting results (like what they report in Fig. 5), and providing to the miRNA field a robust cell-type expression profile for humans. This would eliminate the need for read/rpm cutoffs as they are simply reporting the read profiles for what is in MGDB. This would not hamper their attempts to include these data at UCSC as MGDB includes links to both MB as well as UCSC, and indeed, why report "miRNA" read data to a genome browser for well over a thousand non-miRNAs? This simply will lend credence to all of these non-miRNAs that already clutter the literature. A second paper could focus on potentially interesting or relevant small RNAs that show interesting patterns of expression in normal and/or diseased tissues, highlighting the structural and expression profiles of these genic elements, and possibly trying to identify what they might be (including potential false negatives in MGDB). As Corey and colleagues (2022, NAR) recently stressed, we as a field must focus on mechanism as the identification of a "biomarker" in and of itself is of no real value if we don't understand what it is or where it comes from.
Minor comments:
1) The seed sequence is 7-8 nt in length, not 6 nt.
2) miRNAs reads - both mature and star - have a mean length of 22 nt in length, and no miRNA is less than 20 nt long (5p: median = 22, mean = 22.56, SD = 0.94, range = 20-27; 3p: median = 22, mean = 22.11, SD = 0.57, range = 20-26. All data from MGDG.).
3) Its misleading to write miRNAs "block protein translation." Please rewrite.
4) I don't believe our understanding of the expression profile of miRNAs is hampered by the numerical naming scheme. MB's nomenclature system obscures the evolution of miRNAs by erecting both paraphyletic (e.g., MIR-8, which includes mir-141) and polyphyletic groups. Why would distinct monophyletic families like MIR-142, MIR-143 and MIR-144 create confusion regarding their expression?
5) The use of the term "leading strand" is confusing given its clear association with DNA replication (and not a term I've heard of associated with miRNAs).
6) Please give cut-offs for things like "infrequent", "frequent" etc.
7) I was surprised at the lack of co-expression for Burge's co-targeting miRNAs, especially in the brain. I

think it would be worthwhile to examine more carefully these miRNAs and discuss in a bit of detail why they don't appear together in Fig. 2A.
8) Fig. 6 should be moved to the supplemental figures as this is not readable and of no real value.
9) The authors might want to reference Lu et al. (2005) for Mir-1 expression in the colon as this is one of the obvious down-regulated miRNA in diseased colon tissues.

Methods

Are the methods appropriate to the aims of the study, are they well described, and are necessary controls included? Choose an item.

Conclusions

Are the conclusions adequately supported by the data shown? Choose an item.

Reporting Standards

Does the manuscript adhere to the journal's guidelines on minimum standards of reporting? Choose an item.

Choose an item.

Statistics

Are you able to assess all statistics in the manuscript, including the appropriateness of statistical tests used? Choose an item.

Quality of Written English

Please indicate the quality of language in the manuscript: Choose an item.

Declaration of Competing Interests

Please complete a declaration of competing interests, considering the following questions:

- Have you in the past five years received reimbursements, fees, funding, or salary from an organisation that may in any way gain or lose financially from the publication of this manuscript, either now or in the future?
- Do you hold any stocks or shares in an organisation that may in any way gain or lose financially from the publication of this manuscript, either now or in the future?
- Do you hold or are you currently applying for any patents relating to the content of the manuscript?
- Have you received reimbursements, fees, funding, or salary from an organization that holds or has applied for patents relating to the content of the manuscript?

- Do you have any other financial competing interests?
- Do you have any non-financial competing interests in relation to this paper?

If you can answer no to all of the above, write 'I declare that I have no competing interests' below. If your reply is yes to any, please give details below.

I declare that I have no competing interests

I agree to the open peer review policy of the journal. I understand that my name will be included on my report to the authors and, if the manuscript is accepted for publication, my named report including any attachments I upload will be posted on the website along with the authors' responses. I agree for my report to be made available under an Open Access Creative Commons CC-BY license (http://creativecommons.org/licenses/by/4.0/). I understand that any comments which I do not wish to be included in my named report can be included as confidential comments to the editors, which will not be published.

Choose an item.

To further support our reviewers, we have joined with Publons, where you can gain additional credit to further highlight your hard work (see: https://publons.com/journal/530/gigascience). On publication of this paper, your review will be automatically added to Publons, you can then choose whether or not to claim your Publons credit. I understand this statement.

Yes Choose an item.
