# Supplementary material for: A curated human cellular microRNAome based on 196 primary cell types
Source: Gigascience. 2022 Aug 25;11:giac083. doi: 10.1093/gigascience/giac083 (PMC9404528; doi:10.1093/gigascience/giac083)
Supplement: giac083_Response_to_reviewers_gigasci_v2 [file giac083_response_to_reviewers_gigasci_v2.docx]

July 1, 2022

Dear Dr. Zauner,

Thank you for the email and opportunity to respond to the reviewers. Consistent with my superb experience with your editorial staff, your reviewers made excellent points that have improved the manuscript. Below is a point by point response to the reviewers concerns. We hope these changes are sufficient to satisfy the reviewers, but if not, we are happy to make additional corrections, as needed.

Sincerely,

Marc Halushka

Reviewer reports:

*Reviewer #1: In this paper, the authors reported a curated human cellular microRNAome based on 196 primary cell types. This could be a valuable resource. The following comments could improve this study.*

*1. Euclidean distance could be not a good metric for clustering analysis. I am wondering the results when using other metrics, e.g. spearman's correlation.*

Our goal was to demonstrate the effect of normalization procedures on classification of a test sample to cell-type centroids with already defined labels. The use of Euclidean distance was a more strict assessment of normalization procedures. A Spearman correlation could allow us to classify a sample to a centroid that is highly correlated (similar genes being highly expressed, similar genes being lowly expressed), while the absolute distance in high dimensional space could be quite large (gene expression of the set of highly expressed genes in the test sample could be very different level than gene expression in those genes in the centroid). That being said, we reran the analysis using 1-Spearman correlation and obtained very similar results. In the manuscript, we have left the analysis as determined by Euclidian distance.

|  | Euclidian distance | Spearman  correlation |  |
| --- | --- | --- | --- |
| Accuracy | 0.865 | 0.856 |  |
| Sensitivity by cell type | 0.8 | 0.8 | cardiac muscle |
|  | 0.95 | 0.95 | endothelial |
|  | 0.73 | 0.85 | fat |
|  | 0.8 | 0.79 | lymphocyte |
|  | 0.92 | 0.88 | macrophage |
|  | 1 | 0.79 | smooth muscle |

*2. More analysis are suggested, such as cell-specific miRNA, functional set analysis etc.*

Thank you for the suggestion. Although this study is designed to make miRNA expression patterns of numerous cell types available in one setting, we are open to performing more analysis of these miRNA patterns. Regarding functional set analysis, we considered looking at the most abundant miRNAs by cell type for pathway analysis using Gene Ontology tools. The standard Pantherdb.org tool does not assay miRNAs. We investigated this further and came across GOmir (Roubelakis MG et al, BMC Bioinformatics 2009) as a tool to work with miRNAs. Unfortunately, the tool is no longer available. As we started to look at other GO methods we became concerned about running this experiment due to the biases in gene ontology studies (Fridrich A et al, Bioessays, 2019). This is partly a result of the source of miRNA functional data being primarily oncology based and other inherent issues with inaccurate regulatory assignments of some miRNA-mRNA pairs. We think it is better to not do this experiment than provide inaccurate data analysis.

Instead, we have added a new analysis on miRNA expression abundance based on the evolutionary age of the miRNA, as recommended by reviewer 3. This is described in more detail below.

*Reviewer #2: In this study, Patil and co-workers have combined the largest set of publicly available small RNA-seq datasets to provide a comprehensive analysis of cell-type-specific miRNA distributions. Moreover, the authors made their results easily accessible to the public via Bioconductor and UCSC genome browser. This deeply curated resource is a valuable asset to biomedical research and will help researchers better understand and utilize the otherwise overwhelming number of small RNA-seq datasets currently available.*

*Here are some minor points for the authors to address:*

*1. In the background section, the first sentence, "microRNAs (miRNAs) are short, ~18-21 bp, critical regulatory elements that block protein translation". Mature miRNA is single-stranded, so it would be more appropriate to use 'nt' (nucleotides) instead of 'bp' (base pairs) to describe miRNA length. Additionally, many mature miRNAs have a length of 22 and 23nt. Finally, "block protein translation" is not quite right as mammalian miRNAs are believed to primarily function by promoting the degradation of targeted mRNAs.*

We have corrected the first sentence to read “microRNAs (miRNAs) are short, ~22 nt, critical regulatory elements that represses protein translation and promote degradation of mRNA.” We specifically took the MirGeneDB2.1 *Homo sapiens* mature miRNAs and measured their lengths. Consistent with your suggestion, the median value was 22 and the range was between 20 and 26. We added in “promote degradation of mRNA” and elected to continue to say “represses protein translation” as many notable miRNA authors (Ambros, Bartel, etc) use the term “translational inhibition (Duan Y. et al Cell Reports, 2022, Bartel DP Cell, 2018).

*2. In Fig. 1C, is the "co-dominant" category bar missing? Since the sum of 5p and 3p bars are not equal to 100%.*

Yes, you were correct. We somehow lost the “co-dominant” bar. We have also recalculated dominance in light of comments from Reviewer 3. We have added back the co-dominant bar and it represents 11% of all miRNAs in the new analysis.

*3. In Fig. 1D and 1E, the y-axis label "Unique miRNA count" is misleading/confusing. Would a more appropriate label be "Unique miRNA species"?*

“Species” is a better term and we have made that revision to the label.

*4. In the "DESeq2 VST provided superior normalization" section, the authors mentioned that "An HTML interactive UMAP with cell type information is available in the GitHub repository https://github.com/mhalushka/miOme2/UMAP/Figures)." However, the provided link is not accessible.*

We apologize for that. The correct link is “<https://github.com/mhalushka/miROme/tree/main/data/UMAP/Figures>” and has been corrected in the manuscript.

*Reviewer #3: Patil et al. report the expression profiles of miRNAs and a vast array of other, non-miRNA sequences, across 196 cell types based on thousands of publicaly available data sets. Although this will be an outstanding contribution to the miRNA field, I am recommending rejection for now with the strong encouragement to resubmit once the authors have addressed my concerns. To be frank, the authors have two diametrically opposed research agendas here: 1) What are the expression profiles of bona fide miRNAs (as determined by MirGeneDB); and 2) What else might be expressed in human cell types that could be of interest to small RNA workers and clinicians? Because of these opposed goals, the paper is not only confusing to read and process, but it gives fodder to the numerous paper-mill products that continue to identify non-miRNAs as diagnostic, prognostic, and even mechanistic indicators into virtually every human malady under the sun.*

The reviewer is absolutely correct that we tried to cover two concerns at once. MirGeneDB is the appropriate standard for bona fide miRNAs, which any serious miRNA researcher should be using. This is 100% true for any regulatory/functional studies of miRNAs. However, a good number of studies in the general miRNA area represent a search for a useful disease biomarker. In that capacity, it wouldn’t matter if a transcript of ~22 bp in length was or was not a bona fide miRNA, only that it consistently denoted individuals with a disease of interest. For example, mmu-miR-721 is not a bona fide miRNA and has no human homolog, but in mice, it could to be a specific marker of myocarditis (Blanco-Dominguez R et al NEJM 2021). It would be better that a robust RNA biomarker be discovered, that could be renamed to a non-miRNA term, than have it not evaluated for lack of incorrect nomenclature. In that capacity, identifying small transcripts with specificity to a cell type, could be of value to a set of biomarker researchers. On the other side of that argument, despite thousands of “miRNA biomarker” papers listing every single real and false miRNA as a biomarker of something, most of these correlations are bogus or non-specific miRNA changes. And frequently, for the non-bona fide miRNAs, it is a result of a research group investigating the noisy part of the data. So a key point of this manuscript was to highlight the general non-expression of non-bona fide miRNAs. By doing the alignment in miRBaase, we were able to point out that we had expression coverage of 89.8% of miRBase miRNAs and 99.6% of MirGeneDB2.1 miRNAs. If we had aligned all of the samples with the MirGeneDB exclusively, we would have not been able to make that critical point and this paper and its accompanying UCSC Genome Browser track could not be used to push back on that more dubious science. With this data, any reviewer can postulate what cell related to the disease described might have a change in the proposed miRNA biomarker and, as a first step, verify the miRNA is or is not reasonably expressed. Thus, we used miRBase for alignment. But, just as importantly, we trimmed our analyses down to essentially using only the bona fide miRNAs found in MirGeneDB. We discussed these points in the original discussion, and covered how we did analysis on MirGeneDB miRNAs in the methods section, but I imagine the reviewer was concerned throughout the results section and felt our rationale was too late in the manuscript. As a result, we have moved MirGeneDB descriptors into the paper and more clearly explained where we used MirGeneDB specific miRNAs in the analyses within the results section.

*Let me try and highlight why the use of only MirGeneDB (MGDB) would be highly useful for this paper.*

*1) miRBase (MB) is not consistent in its identification of both arms of a bona fide hairpin, resulting in the authors not reporting star reads for highly expressed miRNAs such as mir-206 and mir-184. Further, there are numerous examples where the authors do in fact report a "mature" versus "star" read without both arms annotated in MB with some included in MGDB (e.g., Mir-944, Mir-3909) and others not (e.g., mir-3615) raising the question of how these data were annotated.*

The reviewer is correct that there are differences between miRBase and MirGeneDB, particularly with the coverage of star reads. As we discuss directly below, passenger strand (star) reads are ~4.2% of all reads. Specific to miR-206 (Hsa-Mir-1-P2_5p*) and miR-184 (Hsa-Mir-184_5p*), we reran samples SRR1055961, SRR1055962, SRR8478258, and SRR8478259 in miRge3.0 for alignment directly to MirGeneDB. These were chosen as they had the highest levels of the guide miRNAs miR-206 (3p) or miR-184 (3p). For each sample, there were no reads for Hsa-Mir-1-P2_5p* and Hsa-Mir-184_5p*, despite the abundance of guide reads. Therefore, by aligning to miRBase, we did not lose significant reads to star miRNAs not present in miRBase. That is actually somewhat expected, as star reads that are not present in miRBase are likely not present there due to a paucity of aligning reads that could have led to their discovery.

*2) The authors write that, "the majority (46%) of the reads are mature miRNAs." But MB makes no attempt to distinguish mature from star arms. Hence, if they are annotating to MB, they cannot distinguish between these two processing products. This is not only confusing, but also very unfortunate as one cannot get a sense of the expression of evolutionary intended gene products versus processing products.*

As we discuss in point minor comment 5 below, there are numerous terms for the two final miRNAs that emerge after DROSHA and DICER cutting of the hairpin. When we used the term “mature miRNAs” we were referring to all miRNAs after the hairpin step. That has been a standard terminology in the miRNA field, and the terminology of miRBase. The reviewer (and MirGeneDB) is using the term “mature” to indicate the non-star (or non-passenger) miRNA. As noted below, we were using the term “leading” (but are changing to “guide”) to reflect the non-star strand. For MirGeneDB-exclusive analyses, we have tried to now use “mature” while pointing out this is the MirGeneDB term for the guide strand. Thus, there is a small terminology divide that has led to some confusion. The 46% is accurate in that that is the percent of aligned reads that are final miRNAs. For the sake of the reviewer, we have further broken out the star miRNAs and determined these represent 4.2% (~394 million) of the >9 billion reads. We have changed this (~46%) sentence to now read “… where the majority (~46%) of the reads are mature miRNAs (~9 billion), of which ~95.8% are guide miRNAs.”

*3) The authors report on the use of 5p versus 3p strand dominance, but have no examples of "co-dominant" miRNAs (Fig. 1C) when, in fact, there are numerous examples in their data including Mir-324, Mir-300, Mir-339, Mir-361 etc. with some switching arms depending on the variant. All of this is available at MGDB; none at MB.*

Our apologies for this mistake. As described above with reviewer 2, we somehow lost the bar for co-dominant miRNAs and we failed to provide the numbers. Our original values were 181 (5p), 168 (3p) and 13 (co-dominant) based on just summing total reads of all miRNAs with ≥1000 total reads. We are now limiting ourselves to MirGeneDB only miRNAs with ≥1000 total reads in ≥100 cells. We then investigate arm switching and any miRNA with <75% of cells expressing the dominant 5p or 3p arm are listed as co-dominant. The new data is now across 203 miRNAs and these are 96 (5p), 84 (3p) and 23 (co-dominant) miRNAs. The co-dominant miRNAs are seen below.

| hsa-miR-106b | hsa-miR-212 | hsa-miR-369 | hsa-miR-485 |
| --- | --- | --- | --- |
| hsa-miR-126 | hsa-miR-324 | hsa-miR-374a | hsa-miR-493 |
| hsa-miR-1307 | hsa-miR-330 | hsa-miR-382 | hsa-miR-574 |
| hsa-miR-144 | hsa-miR-335 | hsa-miR-423 | hsa-miR-708 |
| hsa-miR-151a | hsa-miR-339 | hsa-miR-424 | hsa-miR-873 |
| hsa-miR-193a | hsa-miR-361 | hsa-miR-455 | |

*3) MB does not allow the identification of loop or offset reads separate from the arm reads, allowing to authors to accurately report the amount of reads derived from the "hairpin" versus the arms (and how the authors reported this in Fig. 1B is not at all clear given that these sequences are not annotated as such at MB).*

The reviewer is correct that offset reads are characterized in MirGeneDB and not in miRBase. However, it is the functionality of the alignment software, not the database, that ultimately determines how a miRNA is characterized and counted. miRge3.0 was designed to maximally capture all isomiR reads to any given miRNA. To accomplish this, it iterates the Bowtie alignment steps against a hand-curated set of libraries. Specific for the miRNA libraries is that it includes 2 nucleotides to the start of the 5’ end and 6 nucleotides on the 3’ end of all 5p and 3p mature/star miRNAs. miRge3.0 then characterizes all of the isomiRs and 5’ starting locations using the mirGFF3 format (Desvignes T, et al. Bioinformatics 2020). The only way to differentially characterize offset reads is to consider them as 5’ isomiRs as captured by the mirGFF3 format. And these would be captured identically whether aligned to miRBase of MirGeneDB.

miRge3.0 also specifically captures hairpin reads. After a first alignment of all reads in a FASTQ file to the hand-curated mature (guide + passenger) miRNAs allowing no mismatches, all of the remaining reads are aligned to the hairpin reads of the miRNA, allowing a single mismatch. This step will capture only rare reads that are not in the mature/star miRNA location in the hairpin. This has been documented in all iterations of miRge (Baras AS et al PLOSone 2015; Lu Y et al BMC Bioinformatics 2018; Patil AH et al NAR Genom Bioinform 2021).

To be clear, for this cellular microRNAome, we used our hand-curated mature miRNA library based on the miRBase mature.fa file and the miRBase hairpin.fa file for the hairpin alignments. Thus, we were able to capture both offset and loop reads through the methodology of miRge3.0.

*4) The authors bias their genic origins of small RNA reads by filtering first using MB, and then identifying remaining reads as arising from other sources including tRNAs, rRNAs, mRNAs etc. However, numerous "miRNAs" in MB arise from these genic sources including mir-484 (mRNA) and mir-3648 (rRNA). So if I understand the authors pipe-line these sequences are mistakenly included in the "mature miRNA" column.*

This is partly true. There are many more miRNAs listed in miRBase than MirGeneDB, some of which are clearly not bona fide miRNAs. Many overlap repeat elements and the two listed here (miR-484 and miR-3648) are pieces of other RNA species, as the reviewer indicates. Thus a small subset of the >9 billion reads are from dubious miRNAs not in MirGeneDB. We determined this to be 43,486,771 out of 9,400,736,242 reads listed in current Supplementary Table S4 (0.5%). This number is likely a small percentage as miRge3.0, more than any other miRNA alignment tool we are aware of, minimizes these non bona fide miRNAs. What we have known, going back to the earliest iteration of miRge, is that dubious miRNAs tend to have the majority of their reads be non-templated reads (ie. do not match the genomic sequence). Thus, we incorporated steps into miRge to tackle this. In miRge3.0, we will remove any miRNA for which >90% of its reads were not exact matches to the genomic template (even accounting for known SNPs). These non-templated reads are all acquired in the final isomiR capture step. The setting for this is –ex and we used the default value of 0.1. This results in 0 reads reported for miRNAs that have >90% isomiR reads acquired in the final bowtie alignment step (the data used for this manuscript). While this approach may modestly reduce read counts for some non-miRNAs, we do not consider this a concern for the aims of this manuscript.

*5) The use of MGDB would allow the user to see the saturation of mature reads across the different cell types in Fig. 1E, and, if mature is distinguished by star, then one could also see the (near)-saturation of star reads as well. As it stands, their plot just simply highlights the non-genic nature of much of MB. Further, because MGDB identifies the age of each miRNA, if the authors were interested, they could also test a long-standing pattern that evolutionary older miRNAs are expressed at higher levels than younger miRNAs relative to specific cell types.*

We remade Fig 1E with mature and passenger MirGeneDB miRNAs only. The general structure is unchanged from the prior Fig 1E. We are also including a figure of mature MirGeneDB miRNAs only below. It too is quite similar in structure to the original image and the new Fig 1E, with the main difference being the counts along the Y axis.

The idea to investigate miRNAs by evolutionary age was excellent and we have performed that. Specifically, we investigated 152 mature miRNA expression patterns across 96 cells from 12 cell classes. These 152 miRNAs originated in the Bilateria, Vertebrata, Catarrhini and H. sapiens nodes. We found, as expected, a strong signal that the more ancient miRNAs were expressed across all cell classes. The younger miRNAs were only modestly expressed in sperm and stem cells. This is now seen in Supplementary Figure 12 (below), results on page 10, and methods on page 22.

*6) The authors report the expression profiles of bona fide miRNAs in Figs 3 and 5, but report the expression profiles of non-miRNAs in Fig. 4. These include mir-3150b, mir-4298, mir-569, mir-934, mir-302f, and mir-663b. None of these supposed miRNAs have the requisite reads for miRNA annotation, and all but mir-3150b fail a structural examination as well. In fact, MGDG has no reads (which includes numerous data sets from the Halushka lab) for mir-302f, mi-4298, and mir-569, and only a few reads from one "arm" for mir-663b and mir-3150, highlighting the need to examine these supposed reads in detail. The inclusion of obvious non-miRNAs here is confusing and needlessly undermines the authors study and conclusions.*

This is a fair point. As the reviewer points out, we found almost no bona fide miRNAs that had novel cell type specific patterns. Half of these miRBase miRNAs are not in MirGeneDB which probably speaks to the great strides made with our prior cellular microRNAome. Although, as we discussed above, there could still be value in any small RNA transcript that can serve as a biomarker. Nonetheless, consistent with the reviewer’s concern, we have removed the miRNAs listed here from Fig. 4 that are not found in MirGeneDB.

*So, my strong recommendation is to potentially write two papers. The first (this one here) focuses only on the expression of miRNAs, emphasizing really interesting results (like what they report in Fig. 5), and providing to the miRNA field a robust cell-type expression profile for humans. This would eliminate the need for read/rpm cutoffs as they are simply reporting the read profiles for what is in MGDB. This would not hamper their attempts to include these data at UCSC as MGDB includes links to both MB as well as UCSC, and indeed, why report "miRNA" read data to a genome browser for well over a thousand non-miRNAs? This simply will lend credence to all of these non-miRNAs that already clutter the literature. A second paper could focus on potentially interesting or relevant small RNAs that show interesting patterns of expression in normal and/or diseased tissues, highlighting the structural and expression profiles of these genic elements, and possibly trying to identify what they might be (including potential false negatives in MGDB). As Corey and colleagues (2022, NAR) recently stressed, we as a field must focus on mechanism as the identification of a "biomarker" in and of itself is of no real value if we don't understand what it is or where it comes from.*

Although we appreciate the sentiment of the reviewer and his obvious concerns regarding miRBase, splitting this manuscript into two papers is beyond what is reasonably feasible. We were specifically guided by this comment “Our editorial board member and I feel it is NOT necessary to split the paper into two, and we hope you can address the comments by explicitly mentioning the different types of small RNAs in the data base and the paper.” Thus, instead we have tried to incorporate as much of MirGeneDB as possible into the current manuscript. We now use both miRBase and MirGeneDB naming convention at each mention of a miRNA in the manuscript. We have included a sentence in the introduction that reads “Although the alignments were performed to miRBase v22, most analyses were performed using only the MirGeneDB 2.1 bona fide miRNAs (mature and star).” We also have included a new Supplementary Table 3 that serves as a key to allow any reader to cross reference the two database terms for a given miRNA, which also indicates which MirGeneDB miRNAs were not aligned. We hope with all of the additional explanations and revisions, this version of the manuscript will be acceptable.

Minor comments:

*1) The seed sequence is 7-8 nt in length, not 6 nt.*

We have revised this to say the seed sequence is 7-8 nt in length, although classically it was described as 6bp (Lewis BP et al Cell, 2005; McGeary SE et al. eLife 2022).

*2) miRNAs reads - both mature and star - have a mean length of 22 nt in length, and no miRNA is less than 20 nt long (5p: median = 22, mean = 22.56, SD = 0.94, range = 20-27; 3p: median = 22, mean = 22.11, SD = 0.57, range = 20-26. All data from MGDG.).*

We have made that correction as described above (reviewer 1).

*3) Its misleading to write miRNAs "block protein translation." Please rewrite.*

We see that this concept could be described more clearly. David Bartel’s group recently wrote “Upon processing, miRNAs associate with an Argonaute (AGO) protein and base-pair to sites within mRNAs to direct the destabilization and/or translational repression of these mRNA targets.” (McGeary SE et al. eLife 2022). More consistent with that wording, we have changed this sentence to now read “microRNAs (miRNAs) are short, ~22 nt, critical regulatory elements that repress protein translation and promote degradation of mRNA.”

*4) I don't believe our understanding of the expression profile of miRNAs is hampered by the numerical naming scheme. MB's nomenclature system obscures the evolution of miRNAs by erecting both paraphyletic (e.g., MIR-8, which includes mir-141) and polyphyletic groups. Why would distinct monophyletic families like MIR-142, MIR-143 and MIR-144 create confusion regarding their expression?*

For someone as well-versed in miRNA as this reviewer, it is not a problem. Also, from an evolutionary perspective, MirGeneDB corrects the limitations of miRBase. However, for a non-miRNA researcher who may be dabbling with some miRNA data, it is a problem. Specifically it is the anonymity of the numbers. There are no clues to location or function to miRNAs, whereas genes have more descriptive names such as *MSR* (macrophage scavenger receptor), which denotes a cell type and a function. Thus, for the uninitiated, there is little recognition that MIR-142 (immune cell enriched), MIR-143 (mesenchymal cell enriched) and MIR-144 (red blood cell exclusive) will have vastly different expression patterns across cells. That is the reason we wrote: “There is general anonymity of miRNAs in which the numerical naming scheme (ex. miR-141, miR-142, miR-143, miR-144) hides marked differences in expression patterns and function.”

*5) The use of the term "leading strand" is confusing given its clear association with DNA replication (and not a term I've heard of associated with miRNAs).*

While “leading strand” has been used (Meijer HA et al. Biochem Soc Trans, 2014; Jazdzewski K et al. PNAS 2009; Hasakova K et al, Physiol Res 2017), we agree with the reviewer that it is not the most common terminology. As the reviewer knows, many terms have been used for the non-passenger miRNA, which gets loaded into AGO. These include “guide,” “mature,” “dominant,” “sense,” and “leading.” We have changed this to be “guide strand” in the manuscript when referring to miRBase data.

*6) Please give cut-offs for things like "infrequent", "frequent" etc.*

In the methods we had originally written:

Expression patterns were classified into 5 groups. “Cell specific/near specific” indicated a miRNA in which expression was present in <5 dominant cells based on relative RPM peaks. “Infrequent” indicated a miRNA in which expression was present in ~5-10 dominant cells based on relative RPM peaks. “Frequent” indicated a miRNA present in ~10-30 cell types based on dominant RPM peaks. “Near ubiquitous” was a miRNA with common expression in up to 178 cell types (<90%) at ≥100 RPM. “Ubiquitous” was a miRNA with common expression in >178 cell types (>90%) at ≥100 RPM with no dominant expression patterns. Not all miRNAs were easily placed in a category.

We have tweaked this to indicate the lower threshold for “near ubiquitous” to be “with common expression in ~30-178 cell types.” All other parameters had cut-offs. As noted in the methods “not all miRNAs were easily placed in a category.” For example, if a cell type was expressed in 32 cell types at ≥100 RPM, but most of those cells were closely related (ex. fibroblasts), then that miRNA would be better described as frequent, than near ubiquitous. Thus the “~” was used to denote these were not hard (and ignorant of the biology) cut offs.

*7) I was surprised at the lack of co-expression for Burge's co-targeting miRNAs, especially in the brain. I think it would be worthwhile to examine more carefully these miRNAs and discuss in a bit of detail why they don't appear together in Fig. 2A.*

This is a very good point that the reviewer raised. The Burge paper (Cherone JM et al Genome Res, 2019) investigates co-targeting of miRNAs in brain. They specifically use data from an earlier manuscript of ours (McCall MN et al, Genome Res, 2017) and investigate miRNAs with high co-expression in the brain tissue. As an example, they identify miR-137/miR-138 as being co-expressed. We went back to our data, and indeed, for brain tissue, that is true.

However, among the cellular “neuron” data in the current manuscript, we did not find the same co-expression pattern.

The challenge of cell-specific miRNA data, which we commented on in the original discussion is the culture conditions.

“As most of these cells are grown in culture, this improved cellular microRNAome still suffers from ex vivo alterations in cell expression patterns. New methods to identify in vivo expression patterns may change absolute expression estimates substantially [20-22].”

Our citation 21 is the Kuosmanen SM et al Sci Rep 2017 manuscript which shows marked changes in miRNAs as they passaged out endothelial cells from a fresh harvest. We expect cell culture to drive miRNA expression changes at the absolute level and we hope to overcome this in the future with a technique we are developing in the laboratory to capture cell-specific *in vivo* expression patterns of miRNAs (citation 20).

Specific to the brain/neurons this was an even greater challenge. Neurons are not easily cultured and all of the neurons listed were passaged from different stem cells (NSC, hiPSC, ES) from 14 to 115 days, which would make them akin to primitive neurons. Thus, it is likely that *ex vivo* neuron estimates are particularly less representative of *in vivo* estimates than most cell types. It also explains why brain cells and stem cells are close to each other in the UMAP of Fig 2 as they likely have features of both cell types. In Fig 3A (which we think the reviewer was referring to, instead of Fig 2A), we did not include all miRNAs with specificity for each cell type and only listed miR-9 for neuron. In the figure above, the (stem cell derived) neurons (above the red bar) displayed high levels of miR-9 (by RPM), while the only other cells with elevated miR-9 were some stem cells (past number 2000).

All of this is to say that our neuron cell data does not demonstrate the same co-expression patterns as the tissue data for miR-137/138, for reasons which we believe relate to the more primitive cultured neuron miRNA signals. To specifically address this in the manuscript, we have expanded the section on limitations and added the Burge paper reference.

A continuing limitation of cellular microRNAomes are that the data are collected primarily from cells grown in culture. Ex vivo conditions are known to sometimes dramatically alter miRNA expression patterns, as seen for passaged endothelial cells [20]. Additionally, some cell types, neurons in particular, are cultured only as a derivation from a stem cell. Thus, these cells are likely to be more primitive than fully mature in vivo cell types. In fact, while the neurons in this study all had high levels of miR-9, a classic neural maker, they also shared many miRNAs with stem cells and were notable for being adjacent to stem cells in the UMAP of Fig. 2. The neurons also did not display some of the co-expression patterns of miRNAs described in brain tissue [21]. As most of these cells are grown in culture, this improved cellular microRNAome still suffers from ex vivo alterations in cell expression patterns. New methods to identify in vivo expression patterns may change absolute expression estimates substantially [22, 23].

*8) Fig. 6 should be moved to the supplemental figures as this is not readable and of no real value.*

We have moved Figure 6 to the supplemental figures and it is now Supplementary_Figure_S13.

*9) The authors might want to reference Lu et al. (2005) for Mir-1 expression in the colon as this is one of the obvious down-regulated miRNA in diseased colon tissues.*

The Lu et al paper (Lu J et al Nature 2005), despite being highly cited (>11,000 citations) is problematic. It completely fails to take into consideration cellular composition as a driver of miRNA expression patterns. This is why cell culture lines are so easily classified away from tissues. But across normal tissue vs cancer, this is still true. Since Mir-1 is expressed in mesenchymal cells, it would be expected to be lower in cancer, as the amount of mesenchymal cells are high in normal tissue (colon) but relatively lower in an epithelial-rich tumor (colon cancer). But, Mir-1 is still higher in both compared to a straight epithelial cancer cell line that does not express Mir-1. We cover this extensively in Kent OA et al NAR 2014 (Lessons from miR-143/145: the importance of cell-type localization of miRNAs) and specifically comment on the Lu paper.

Even the landmark study of Lu et al. demonstrating the power of miRNAs to classify tumor types, unknowingly utilized cell-specific miRNAs found in supporting or stromal cells to help segment their cancer data (73).

I’d rather not give that manuscript another citation here, but it does accidentally make the case why cellular, not tissue-based miRNA expression estimates are critical and furthers that this paper needs to be seen by the miRNA community.
